# Cervical Dermal Sinus Tract: A Case Report and Comprehensive Literature Review

**DOI:** 10.7759/cureus.51883

**Published:** 2024-01-08

**Authors:** Cristopher Ramirez-Loera, Víctor Hugo Galván Soto, Ricardo Martínez-Pérez, Armando S. Ruiz-Treviño

**Affiliations:** 1 Neurological Surgery, High Specialty Regional Hospital Bajio, León, MEX

**Keywords:** congenital spinal defects, dysraphism, cervical midline lesion, tethered cord, dermal sinus tract

## Abstract

The cervical and thoracic dermal sinuses are rare entities, conforming epithelium-lined tracts that extend from an opening in the skin through a corridor to the layers of the spinal cord. They are commonly detected in early childhood; however, adult reports are singularly rare, especially in cervical regions.

We report a very unusual case of a 45-year-old Mexican female who developed progressive left-side weakness and dexterity suffered from childhood, getting worse in the last year. Physical examination revealed a soft, congenital round cystic lesion in the dorsal-midline skin at the level of C4-C5 vertebrae with no previous treatment received. MRI showed a dermal sinus tract at the C4 level from the skin tethering to the spinal cord and syringomyelia. CT scan showed a dysraphism corresponding to spina bifida at the C4 level and an incomplete closure at the C3 and C5 vertebrae. We surgically managed the lesion by microscopic resection with C3-C5 laminectomy preserving strength and sensitivity. Follow-up MRI showed no residual lesion and contained fistula with no further complications.

Cervical dermal sinus lesions are unusual entities, even less prevalent in adulthood. It represents a possible delay in diagnosis and an increased rate of complications. Early suspicion of the condition is required to make an accurate diagnosis since it is a potentially treatable lesion with a high risk of sequelae without surgical treatment.

## Introduction

Dermal sinus tract (DST) is a rare entity presented as congenital spinal dysraphism attributable to an early neurodevelopmental failure [1-3]. Incomplete separation during fetal development of dermal appendages from neuroectoderm drives a persistent connection between skin and neural structures lined ultimately by stratified squamous epithelium. This tract formation supports Mount’s observation, which detected central nervous system structures communication by the dermatomal level of the defect [4]. The incidence accounts for one case per 2500 live births [5]. Site of presentation hoard predominantly lumbar and lumbosacral regions by 40% and 45% of all cases, respectively [3]. Cervical and thoracic regions exhibit <1% and 10% of reported cases [5,6]. Frequent clinical presentations include skin abnormalities, infections, and neurological deficits. Neurological signs are caused by the inclusion of tumors or by sinus-related tissue reactions and adhesions that tether the spinal cord [7]. However, most patients come to clinical attention until either infection or neurologic deficit interacts with skin findings generally as symptom constellation [3,8].

This report describes a case of cervical DST in a woman who presented progressive loss of strength and dexterity from childhood, which worsened in the last year. A congenital cystic rounded lesion was found and excised successfully.

## Case presentation

Patient history

A 45-year-old female presented to the clinic with progressive left-sided weakness and dexterity suffered from childhood, which worsened in the last year. Physical assessment revealed a soft, congenital round cystic lesion in the dorsal-midline skin at the level of C4-C5 vertebrae with no previous treatment received. Neurological examination exposed left arm manual muscle testing 3/5, hypoesthesia in C5-C8 dermatomes, altered proprioception, positive Hoffmann and Tromner signs, and hyperreflexia. Left lower extremity presented 4/5 on manual muscle testing and no sensitive alterations.

Neuroimaging and surgical approach

**Figure 1:**
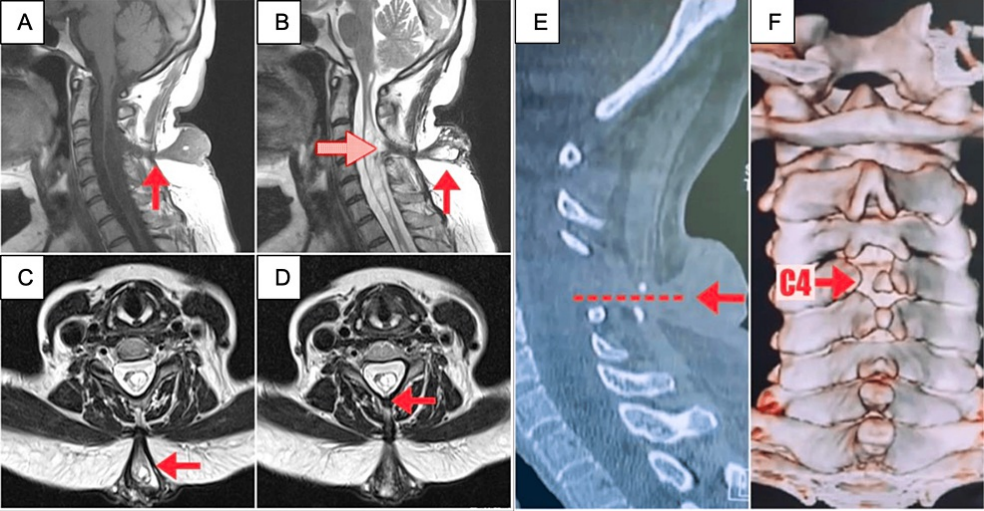
Preoperative imaging (A-F) MRI and CT scan during diagnostic assessment. Sagittal T1 and T2 (A-B) images show the crucial path of the lesion (red arrow) identified from the skin to the spinal cord. Note the dermal sinus extending cranially from the opening lesion. Tethering causes a dorsal displacement of the cord and syringomyelia (dotted red arrow). (C-D) Axial T2 imaging demonstrates the tract crossing all skin layers heading to the spinal cord (red arrows). CT scan (E-F) displayed the conduit trajectory in the sagittal bone window (arrow and dotted line). Coronal CT scan with three-dimensional reconstruction (F); posterior view showing dysraphism of the spinous process and posterior arch of C4 (red arrow). C3 and C5 exhibit dysraphism with small spinous processes and notches allowing abnormal tract continuity.

Magnetic resonance imaging (MRI) showed a DST at the C4 level from the skin tethering to the spinal cord and syringomyelia. T1 and T2 sequences demonstrated an isointense and hyperintense lesion, respectively (Figure 1).

CT scan showed a dysraphism corresponding to spina bifida at the C4 level and an incomplete closure at the C3 and C5 vertebrae (Figure 1). Once identified, we surgically managed the lesion by microscopic resection with C3-C5 laminectomy with screws to lateral masses. After the surgical approach, the patient preserved strength and sensitivity in postoperative outcomes. Subsequent control MRI demonstrated complete resection and containment of the fistula.

Postoperative course

**Figure 2:**
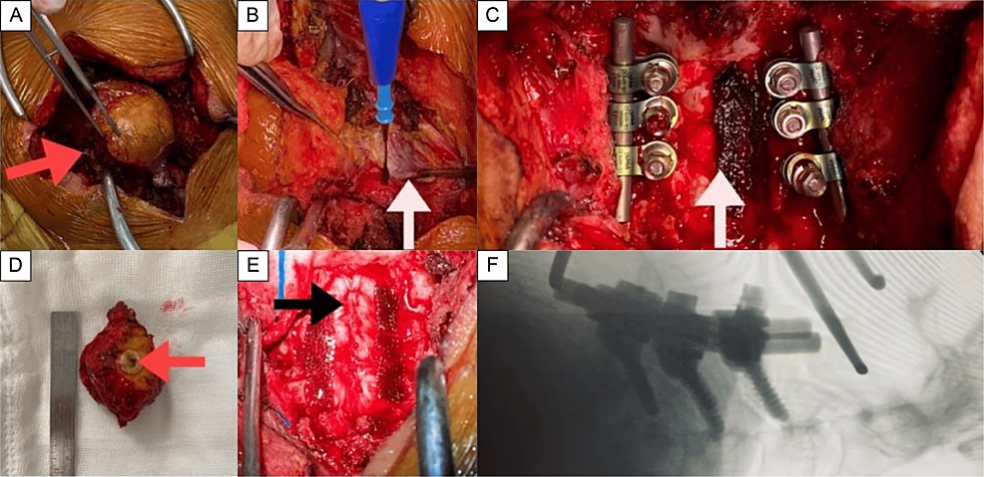
Intraoperative imaging and surgical approach (A-F) Gross imaging and fluoroscopy during surgical resection. (A) The tract was followed from the skin to its intradural location without losing the main trajectory (red arrow). (B) Muscle layer trajectory. Although we identified the tract, laminectomy of C3, C4, and C5 was performed to continue a wide and safe exploration (white arrow). (C) Intraoperative view showing posterior cervical instrumentation of C3, C4, and C5 (white arrow). (D) Gross examination of the 5 cm specimen showed a 3 mm diameter lumen of the tract (red arrow). (E) Post-closure capture with reference to the midline (black arrow). (F) Lateral fluoroscopy imaging. Posterior cervical fusion was performed due to multiple cervical vertebrae dysraphism, which prevented performing laminotomy.

The surgery was performed after evaluating the risk of complications primarily derived from this entity (Figure 2).

According to preoperative neurological deficits established, the patient progressively benefited after surgical intervention in the long term. An appropriate surgical technique must be performed, after cautiously suspecting this entity, which is mandatory to be present as a differential diagnosis within the spectrum of neurological deficits.

## Discussion

The first congenital DST was reported in 1865 [9]. However, Walker et al. described a case series of meningitis and pathological fistulous tract formation while documenting several aspects of this rare congenital disease [10]. The cervical region accounts for less than 1% of the cases [2,3]. Therefore, reports are scarce from this unusual entity. One of the main concepts not yet elucidated is about the formation hypothesis of this disease, specifically during the embryogenic period and the three differentiated layers: ectoderm, endoderm, and mesoderm. There is only knowledge of a plausible congenital error in separating the two layers of the superficial ectoderm, cutaneous ectoderm, and neuroectoderm, until the end of neurulation.

Ordinarily, DST is diagnosed during childhood. The diagnosis during adulthood is exceedingly rare, even more so in the cervical region [7,8]. Due to its cervical onset site, it can cause neurological deficits due to tethered cord syndrome [8]. The spinal column matures more promptly than the spinal cord. It may cause congenital DST formation combined with neuroectoderm errors. Symptoms in adult patients may be associated with a tethered cord. Thus, strands of connective tissue trigger tethering to the dorsal dura mater with elongation of the spinal cord at an anteroposterior angle. Also, a normal range of motion of the cervical area leads to elongation of the spinal cord. Diagnosis made by MRI is the standard choice for visualizing a fistulous tract with low intensity in both T1 and T2. Notably, only 40% of the cases are diagnosed on the preoperative MRI scan. If the MRI scan is normal and a skin lesion is identified, suggestive of dermal sinus, surgical exploration of the lesion is appropriate. Authors recommend subdural revision in every case to exclude arachnoid strands causing tethered cord syndrome [1,2].

Undoubtedly, this has also been facilitated by the increased accessibility of sophisticated neuroimaging procedures. Despite skin abnormalities being the primary reason for referral to a neurosurgeon, a considerable proportion of patients develop neurologic deficiency at the time of initial evaluation. These findings demonstrate that delays in primary diagnosis contribute to neurologic sequelae development. When treating patients with DSTs, the primary care physician should initially consider this entity [11-13]. Total excision of the dermal sinus is the primary goal of surgery. To completely remove the dermal sinus and other intradural lesions, surgeons typically need to breach the dura. Exemplified in this case, the dura was opened for the DST, removing and untethering the cord. The main goals for this approach target preventive measurements, for symptomatic tethered cords, or treating acute neurological impairments [14]. Surgical intervention should be performed as soon as feasible to repair the dermal sinus linked to the tethered cord and prevent early neurological decline.

## Conclusions

Cervical dermal sinus lesions are unusual entities, even less prevalent in adulthood. It represents a possible delay in diagnosis and an increased rate of complications. Early suspicion of the condition is required to make an accurate diagnosis since it is a potentially treatable lesion with a high risk of sequelae without surgical treatment. Surgical intervention is conducted as a preventive measure, for symptomatic tethered cords, or treating acute neurological impairments. It is important for the primary care provider to keep several tenets in mind when initially evaluating patients with congenital dermal malformations. A surgical approach should be performed as soon as feasible to repair the dermal sinus linked to the tethered cord toward neurological decline prevention.
